# Supplementary material for: Causal Relationship between Aging and Anorexia Nervosa: A White-Matter-Microstructure-Mediated Mendelian Randomization Analysis
Source: Biomedicines. 2024 Aug 16;12(8):1874. doi: 10.3390/biomedicines12081874 (PMC11351342; doi:10.3390/biomedicines12081874)
Supplement: Supplementary file 1 [file biomedicines-12-01874-s001.zip › Supplementary Figure/Figure S4/Singe SNP plot of MR analysis for TL to ubm-b-1617.pdf]

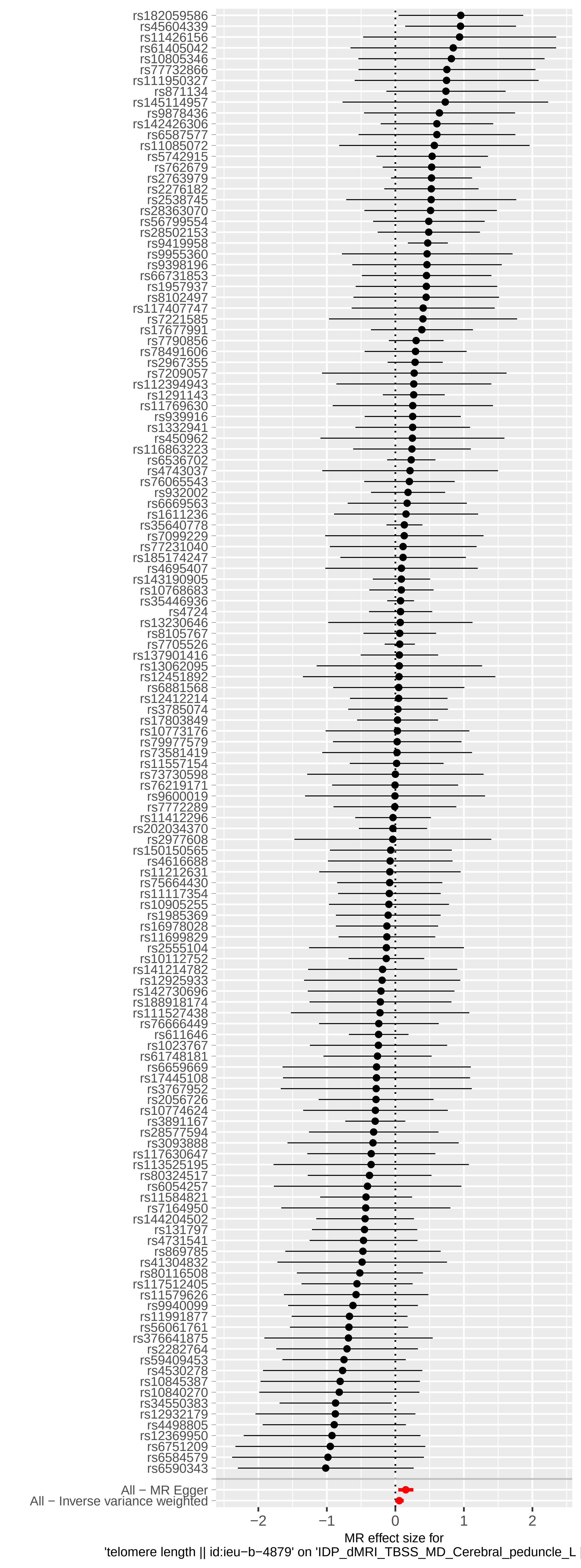
rs182059586
rs45604339
rs11426156
rs61405042
rs10805346
rs77732866
rs111950327
rs871134
rs145114957
rs9878436
rs142426306
rs6587577
rs11085072
rs5742915
rs762679
rs2763979
rs2276182
rs2538745
rs28363070
rs56799554
rs28502153
rs9419958
rs9955360
rs9398196
rs66731853
rs1957937
rs8102497
rs117407747
rs7221585
rs17677991
rs7790856
rs78491606
rs2967355
rs7209057
rs112394943
rs1291143
rs11769630
rs939916
rs1332941
rs450962
rs116863223
rs6536702
rs4743037
rs76065543
rs932002
rs6669563
rs1611236
rs35640778
rs7099229
rs77231040
rs185174247
rs4695407
rs143190905
rs10768683
rs35446936
rs4724
rs13230646
rs8105767
rs7705526
rs137901416
rs13062095
rs12451892
rs6881568
rs12412214
rs3785074
rs17803849
rs10773176
rs79977579
rs73581419
rs11557154
rs73730598
rs76219171
rs9600019
rs7772289
rs11412296
rs202034370
rs2977608
rs150150565
rs4616688
rs11212631
rs75664430
rs11117354
rs10905255
rs1985369
rs16978028
rs11699829
rs2555104
rs10112752
rs141214782
rs12925933
rs142730696
rs188918174
rs111527438
rs76666449
rs611646
rs1023767
rs61748181
rs6659669
rs17445108
rs3767952
rs2056726
rs10774624
rs3891167
rs28577594
rs3093888
rs117630647
rs113525195
rs80324517
rs6054257
rs11584821
rs7164950
rs144204502
rs131797
rs4731541
rs869785
rs41304832
rs80116508
rs117512405
rs11579626
rs9940099
rs11991877
rs56061761
rs376641875
rs2282764
rs59409453
rs4530278
rs10845387
rs10840270
rs34550383
rs12932179
rs4498805
rs12369950
rs6751209
rs6584579
rs6590343
All − MR Egger
All − Inverse variance weighted
−2
−1
0
1
2
MR effect size for
'telomere length || id:ieu−b−4879' on 'IDP_dMRI_TBSS_MD_Cerebral_peduncle_L
